# Placental vascularity and markers of angiogenesis in relation to prenatal growth status in overnourished adolescent ewes

**DOI:** 10.1016/j.placenta.2016.08.076

**Published:** 2016-10

**Authors:** David J. Carr, Anna L. David, Raymond P. Aitken, John S. Milne, Pawel P. Borowicz, Jacqueline M. Wallace, Dale A. Redmer

**Affiliations:** a Rowett Institute of Nutrition and Health, University of Aberdeen, Greenburn Road, Bucksburn, Aberdeen, AB21 9SB, UK; b UCL Institute for Women's Health, University College London, 86-96 Chenies Mews, London, WC1E 6HX, UK; c Department of Animal Sciences, North Dakota State University, Fargo, ND, 58108, USA

**Keywords:** Placental vascularity, Fetal growth restriction, Angiogenic factors, Sheep, Angiopoietin

## Abstract

**Introduction:**

Placental vascularity may be important in the development of fetal growth restriction (FGR). The overnourished adolescent ewe is a robust model of the condition, with ∼50% of offspring demonstrating FGR (birthweight >2 standard deviations below optimally-fed control mean). We studied whether placental vascularity, angiogenesis and glucose transport reflect FGR severity.

**Methods:**

Singleton pregnancies were established in adolescent ewes either overnourished to putatively restrict fetoplacental growth (n = 27) or control-fed (n = 12). At 131d (term = 145d) pregnancies were interrupted and fetuses classified as FGR (n = 17, <4222 g, -2SD below control-fed mean) or non-FGR (n = 10). Placentome capillary area density (CAD), number density (CND), surface density (CSD), and area per capillary (APC) in the fetal cotyledon (COT) and maternal caruncle (CAR) were analysed using immunostaining. COT/CAR mRNA expression of angiogenic ligands/receptors and glucose transporters were measured by qRT-PCR.

**Results:**

Fetal weight was reduced in FGR vs. Non-FGR/Control groups. Total placentome weight was Control > Non-FGR > FGR and fetal:placental weight ratios were higher in overnourished versus Control groups. COT vascular indices were Non-FGR > FGR > Control. COT-CAD, CSD and APC were significantly greater in Non-FGR overnourished versus Control and intermediate in FGR groups. CAR vascularity did not differ. CAR-*VEGFA/FLT1/KDR/ANGPT1/ANGPT2/SLC2A1*/*SLC2A3* mRNA was lower and COT-*ANGPT2* higher in overnourished versus Control groups.

**Discussion:**

Relative to control-intake pregnancy, overnourished pregnancies are characterised by higher COT vascularity, potentially a compensatory response to reduced nutrient supply, reflected by higher fetal:placental weight ratios. Compared with overnourished pregnancies where fetal growth is relatively preserved, overnourished pregnancies culminating in marked FGR have less placental vascularity, suggesting incomplete adaptation to the prenatal insult.

## Highlights

• Overnourishment of adolescent sheep dams produces FGR in approximately 50% of cases.
• Cotyledonary vascularity is increased in overnourished vs. control-intake pregnancy.
• Cotyledonary vascularity is highest in non-FGR cases, suggesting greater adaptation.
• Changes in cotyledonary vascularity are mirrored by angiopoietin-2 mRNA expression.
• Caruncular angiogenic ligands are reduced in FGR without any changes in vascularity.

## 1 Introduction

Fetal growth restriction (FGR), wherein the fetus fails to achieve its growth potential, remains a leading cause of perinatal morbidity and mortality [1]. The most common cause of FGR is uteroplacental insufficiency, in which delivery of oxygen/nutrients to the fetus is limited [2]. The sheep is widely used to model placental insufficiency through premating carunclectomy, placental embolisation, single umbilical artery ligation and maternal hyperthermia, which all adversely impact placental weight/function [3]. FGR may also be induced by overnourishing pregnant adolescent ewes [4]; high dietary intakes in still-growing dams promote nutrient partitioning to maternal tissues, away from the fetoplacental unit, resulting in FGR compared with control-fed adolescent dams. Accordingly, the greater the anabolic drive in the young mother, the greater the birth weight reduction in her offspring; this is highlighted by the fact that maternal gestational weight gain (g/day) in the first third of pregnancy is inversely related to the degree of FGR [5].

The placenta is a key determinant of FGR in this paradigm; placental weight correlates strongly with late-gestation fetal weight and lamb birthweight across studies [5] and although placental weight *per se* is not impacted until ∼d100 (term = 145d), early stigmata of placental dysfunction are evident. Capillary density in the fetal cotyledon (COT) is lower at d50 [6] and mRNA expression of five angiogenic ligands/receptors is attenuated in d81 placentomes [7]. Placental cellular proliferation is lower and secretory function (e.g. placental lactogen) impaired [8], [9], [10]. Serial assessments of uterine blood flow (UBF) and ultrasound markers of placental size reveal reductions in overnourished pregnancies from mid-pregnancy onwards [11], [12]. By the final third of gestation, placental weight is significantly lower, mirrored by reductions in placental glucose transport and fetal nutrient uptakes, ultimately constraining fetal growth [13].

However, not all fetuses of overnourished adolescent dams are markedly growth-restricted at term; historically, only ∼50% of offspring of overnourished mothers have a birthweight >2SD below the mean birthweight of normally-grown controls. The remainder exhibit relatively “normal” birthweight despite equivalent maternal nutritional manipulation and significant shifts in placental weight relative to control-fed dams and are considered “non-FGR”. We hypothesised that differences in placental vascular indices and/or expression of key angiogenic ligands/receptors exist between FGR and non-FGR overnourished pregnancies, and may explain the apparent reserve capacity of the placenta, which maintains adequate fetal growth in a proportion of overnourished pregnancies despite early placental insults.

## 2 Materials and methods

### 2.1 Experimental animals and study design

Animal procedures were approved and regulated by the UK Home Office (Animals (Scientific Procedures) Act 1986) and local ethics committee review. Ewes were housed in individual pens under natural lighting conditions at the Rowett Institute (57^°^N, 2^°^W). Singleton pregnancies with maximum genetic homogeneity were generated using superovulation and laparoscopic intrauterine insemination in donor adult ewes, and embryo transfer into recipient adolescent ewe lambs, as described [14]. Immediately following embryo transfer and throughout pregnancy, ewes were fed a control ration to support normal fetoplacental development (n = 12) or a high intake to potentially restrict fetoplacental growth (n = 27), as described [15].

### 2.2 Ultrasound assessment

At d126 ± 0.3 ewes underwent detailed ultrasound examination including fetal biometry [16]: abdominal circumference (AC), renal volume (RV), biparietal diameter (BPD), tibial length (TL) and femur length (FL). Placentome index was calculated (sum of individual cross-sectional areas of ten representative placentomes). Umbilical cord diameter (UCD) was measured close to the abdominal insertion. Umbilical artery (UA) pulsatility index (PI), resistance index (RI) and systolic:diastolic ratio (SDR) were measured using Doppler waveform analysis [12].

### 2.3 Necropsy and tissue sampling

At d131 ± 0.3, maternal plasma was sampled for the subsequent measurement of metabolic parameters (see below), then ewes were killed by intravenous pentobarbital sodium overdose (200 mg/ml). Fetuses were delivered by hysterotomy and euthanased (same method) before being dried/weighed. The placenta occupied the gravid and non-gravid uterine horn in all cases and placentomes representative of average size and gross morphology (based on the classification system of Vatnick et al. [17]) were sampled from the lower third of the non-gravid horn. A group of six placentomes were weighed, and a size and morphology category attributed: four placentomes were separated into COT and maternal caruncular (CAR) components by gentle traction, snap-frozen in isopentane chilled with liquid nitrogen and stored at −80 °C, and two whole placentomes were sliced into 5 mm cross-sections, immersion-fixed in Carnoy's solution and prepared for immunofluorescence analysis, as described [18], [19]. Remaining placentomes were dissected, weighed and a size and morphology category attributed. On this basis it was established whether the placentomes rapidly selected for snap-freezing/histology were representative of the entire placenta. For 35 of 39 pregnancies the sampled placentomes were entirely representative of the most prevalent gross morphology category observed in the individual. For the remaining four animals, the sampled placentomes were representative by mass but not number because they were very large. Thus, three animals had D type placentomes sampled (all controls) and one had C type placentomes sampled (non-FGR). The total placentome weight was added to the membrane weight to give the total placental weight.

### 2.4 Metabolic parameters

Plasma glucose levels were determined in maternal blood samples using a dual biochemistry analyser (Model 2700, Yellow Springs Instruments, Yellow Springs, OH, USA) and variation between duplicates was <5%. Plasma insulin and insulin-like growth factor (IGF)-1 levels were determined using double antibody radioimmunoassays, as previously described [20], [21]. The limits of sensitivity for the insulin and IGF1 assays were 0.08 and 0.04 ng/ml, respectively, and the intra- and inter-assay coefficients of variation were both <10%.

## 3 Quantitative RT-PCR

RNA was extracted from separately-frozen COT/CAR tissues and subjected to qRT-PCR for nine angiogenic ligands/receptors and two glucose transporters using ovine-specific Taqman probes/primers, as described [7], [22]: *VEGFA, FGF2, NOS3, ANGPT1, ANGPT2, FLT1, KDR, TEK, GUCY1, SLC2A1* and *SLC2A3*. mRNA expression for each gene of interest was quantified using a standard curve based on reference cDNA generated from RNA from pooled d131 placentomes (representative of control and overnourished groups), and normalised to 18S.

### 3.1 Assessment of placental vascularity/proliferation

**Fig. 1:**
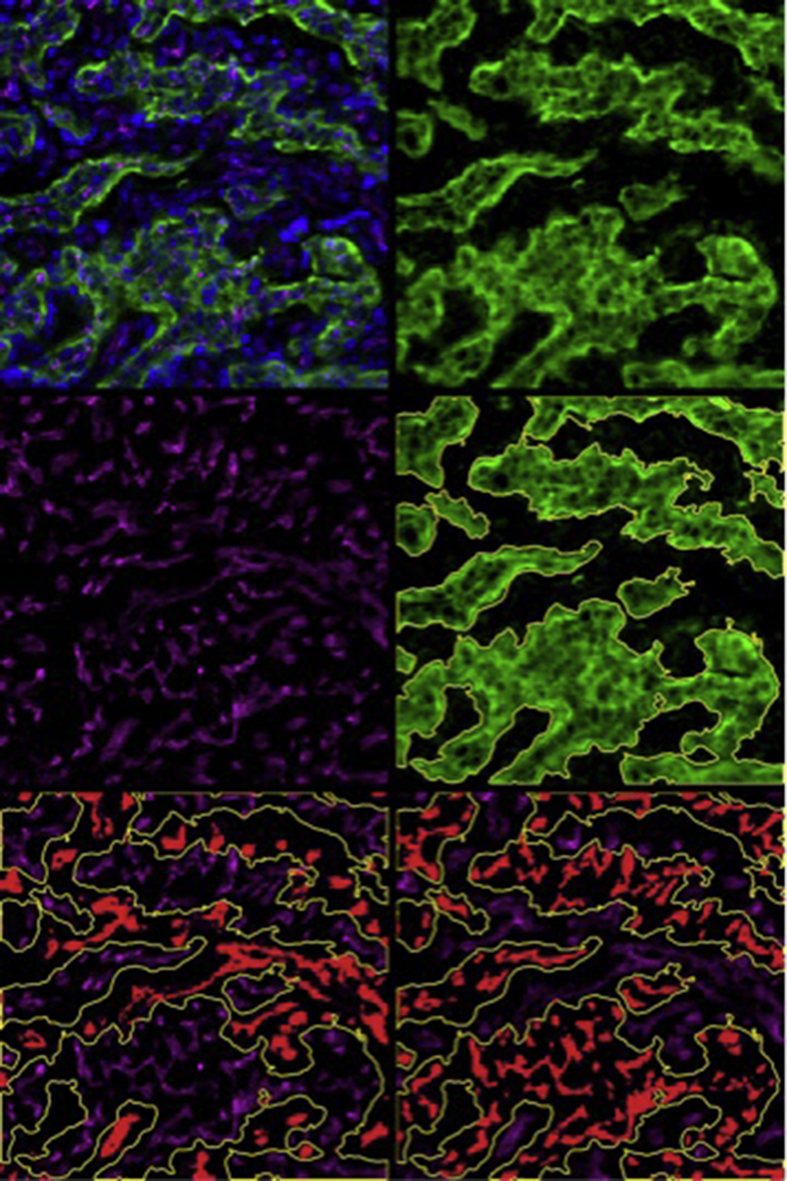
**Representative images of immunofluorescence staining of whole placentomes**. Representative photomicrograph of section of whole placentome triple stained for vascularity, fetal trophoblast and cell nuclei [upper left] sampled from adolescent sheep dams at 131 ± 0.3 days gestation. Magnification = x200. [upper right] Bright green selection demonstrating fetal portion of placentome (cotyledon) based on lectin FITC staining, [middle right] fetal trophoblast circumscribed with ImagePro Premier software for determining fetal (cotyledon) and maternal (caruncle) compartments. [middle left] CD31 staining (magenta) revealing all blood vessels within placentome. [lower left] blood vessels highlighted (red) within maternal portion of placentome for quantification. [lower right] blood vessels highlighted (red) within fetal portion of the placentome for quantification.

Paraffin-embedded 5 μm placentome sections underwent rehydration in ethanol, and antigen retrieval using 50 mM glycine, 1 mM EDTA and 0.05% Tween 20 (pH 9.0) at 120 °C for 10 min. Sections were incubated with anti-CD31 primary antibodies (ab28364, Abcam, Cambridge, UK) and CF633 goat anti-rabbit IgG secondary antibodies (20122, Biotium, Hayward, CA, USA) to identify all blood vessels (maternal/fetal) by immunofluorescent labeling. The trophoblast layer was stained with 20 μg/ml FITC-labeled BS1 lectin (FL-1101; Vector Laboratories, Burlingame CA, USA) and proliferating cells were stained with anti-Ki67 mouse monoclonal antibody (VP-k452, Vector Laboratories), as described [18]. Cell nuclei were counterstained with DAPI (P36931, Life Technologies) for total (nucleus) counts and proliferation reported as percentage of Ki67 stained nuclei. Placental vascularity was quantified, as described [18], [23] and vascularity and cell proliferation reported as mean of four images per section from one placentome per animal. Blood vessels were identified through anti-CD31 immunofluorescence. CAR/COT compartments were identified based on specific lectin staining of fetal trophoblast and individually circumscribed. Capillary number (per mm^2^), perimeter (μm) and area (μm^2^) were determined, from which the following vascular indices were calculated (separately in CAR/COT): capillary area density, CAD (capillary area ÷ tissue area); capillary number density, CND (capillary number ÷ tissue area); capillary surface density, CSD (capillary perimeter ÷ tissue area); and area per capillary, APC (capillary area ÷ capillary number). Representative immunofluorescence images (separately/overlaid) and analyses are presented in Fig. 1 (Ki67 staining not shown).

### 3.2 Statistical analysis

Fetuses of overnourished dams were classified as FGR (n = 17) or non-FGR (n = 10) according to an accepted definition (weight >2SD below genetically-matched control mean [24]). The cut-off was 4222 g. Data were analysed in SPSS v19.0 (SPSS Inc, Chicago, IL, USA). After confirming normality and equality of variance using Q-Q plots and Levene's test, respectively, the three groups (Control, FGR and Non-FGR) were compared using one-way ANOVA and post-hoc test of least significant difference (LSD). The effect of diet alone (control-intake vs. all overnourished) was assessed using Student's *t*-test. Data are presented as mean ± standard error (SEM). Statistical significance was set at p < 0.05.

## 4 Results

### 4.1 Late gestation ultrasound assessment

Table 1 shows ultrasound findings at d126 ± 0.3. By this stage of gestation, all fetal biometry was tracking significantly smaller in overnourished pregnancies subsequently defined as FGR, compared to control-intake pregnancies. Measurements of BPD, AC, FL, TL and RV were larger in Control versus FGR groups (p < 0.001–0.008) and intermediate in the Non-FGR group. For AC/RV (most sensitive growth indices) and UCD, Non-FGR measurements were smaller than controls (p=<0.001/p = 0.017/p = 0.008) but larger than the FGR group (p = 0.02/p = 0.037/p = 0.009). TL/FL were also greater in Non-FGR versus FGR groups (p = 0.001/p = 0.033). Placentome indices did not differ between FGR and Non-FGR groups (p = 0.366) but were smaller in both overnourished groups relative to controls (p < 0.001/p = 0.014). Likewise, UA Doppler indices (PI/RI/SDR) were equivalent in FGR and Non-FGR overnourished pregnancies (p > 0.05), and all were higher relative to control-intake pregnancies (p < 0.001–0.005), indicating higher impedance.

### 4.2 Pregnancy outcome

**Fig. 2:**
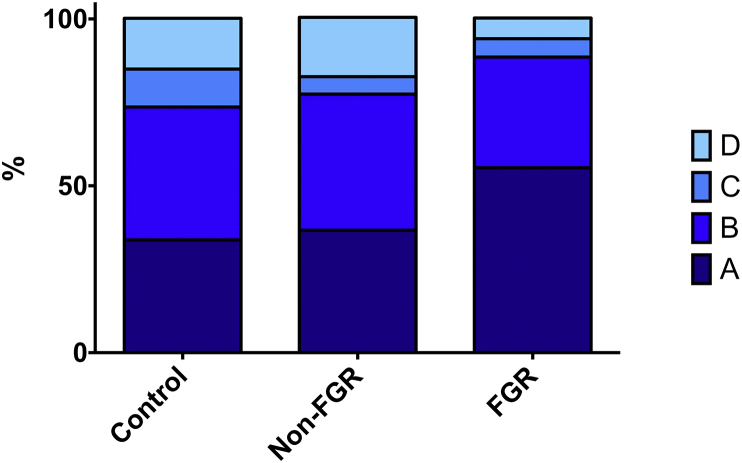
**Placentome types**. Proportions of placentomes types A, B, C and D (according to the classification of Vatnick et al. [17]) sampled from 39 adolescent sheep dams at 131 ± 0.3 days gestation receiving a control intake (Control group, n = 12) or a high intake (overnourished, n = 27) of the same complete diet to induce normal fetoplacental growth or placental and fetal growth restriction (FGR), respectively. Overnourished pregnancies were further subdivided into those subsequently defined as FGR, (fetal weight >2SD below Control group mean, n = 17) or less perturbed with relatively “normal” fetal weight (Non-FGR, n = 10).

Table 2 shows pregnancy outcomes at d131 ± 0.3. By design, FGR fetuses weighed less than Control and Non-FGR fetuses (28%/25%, p < 0.001) whilst Control and Non-FGR groups did not differ. Total/average placentome and total placental weights were reduced in FGR and Non-FGR groups compared with controls (p = 0.002–0.01). Although placentome number was equivalent, the total placentome/placental weight was attenuated in FGR pregnancies compared with the two other groups (p < 0.01). The proportion of placentomes ranging between 1 and 2 cm tended to be significantly greater in FGR versus Control groups (p = 0.06). Although there were no statistically significant differences between the three groups with respect to placentome type, there was a trend towards a greater proportion of A type and smaller proportion of D type placentomes in the FGR group (Fig. 2). The proportion of A type placentomes was inversely correlated with CAR APC (r = −0.415, n = 39, p = 0.015). Fetal:placental weight ratios did not differ between overnourished subsets (p = 0.618), but were greater in FGR/Non-FGR groups versus controls (p = 0.018/p = 0.029). Taking all overnourished pregnancies together, fetal:placental weight ratios were greater than in control-intake pregnancies (11.8 ± 0.43 vs. 9.9 ± 0.42, p = 0.01). Fetal and total placental weights correlated strongly in overnourished (r = 0.787, n = 27, p < 0.001) but not control-intake (r = 0.365, n = 12, p = 0.243) pregnancies. With respect to maternal metabolic status, compared to the Control group, mothers of FGR and Non-FGR overnourished offspring demonstrated higher plasma levels of glucose (69.1 ± 1.52 and 69.9 ± 2.05 vs. 62.3 ± 1.44 mg/dl, respectively, p = 0.006), insulin (2.44 ± 0.173 and 1.79 ± 0.135 vs. 1.13 ± 0.157 ng/ml, respectively, p < 0.001) and IGF1 (0.49 ± 0.023 and 0.41 ± 0.023 vs. 0.36 ± 0.0217 ng/ml, respectively, p = 0.001).

### 4.3 Placental mRNA expression

Table 3 shows expression of angiogenic ligands/receptors and glucose transporters in the fetal cotyledonary (COT) and maternal caruncular (CAR) tissues. In the CAR, mRNA expression of seven of 11 parameters was markedly reduced in FGR overnourished versus control-intake pregnancies. Thus, values were reduced in FGR versus Control groups for *VEGFA* (p = 0.001), *FLT1* (p = 0.001), *KDR* (p = 0.003), *ANGPT1* (p = 0.007), *ANGPT2* (p = 0.023), *SLC2A1* (p = 0.004) and *SLC2A3* (p = 0.025). In general, values in the Non-FGR overnourished group were intermediate and did not significantly differ from the Control group with the exception of *SLC2A3*, which was expressed less (p = 0.001). For five CAR parameters, there was greater expression in Non-FGR versus FGR group: *FLT1* (p = 0.007); *KDR* (p = 0.042); *ANGPT1* (p = 0.003); *ANGPT2* (p = 0.005); and *SLC2A1* (p = 0.003). By contrast, there were no significant differences in the COT with the exception of *ANGPT2*, expression of which was greater in both FGR/non-FGR overnourished groups relative to controls (p = 0.031/p = 0.003).

### 4.4 Placental vascularity/proliferation

**Fig. 3:**
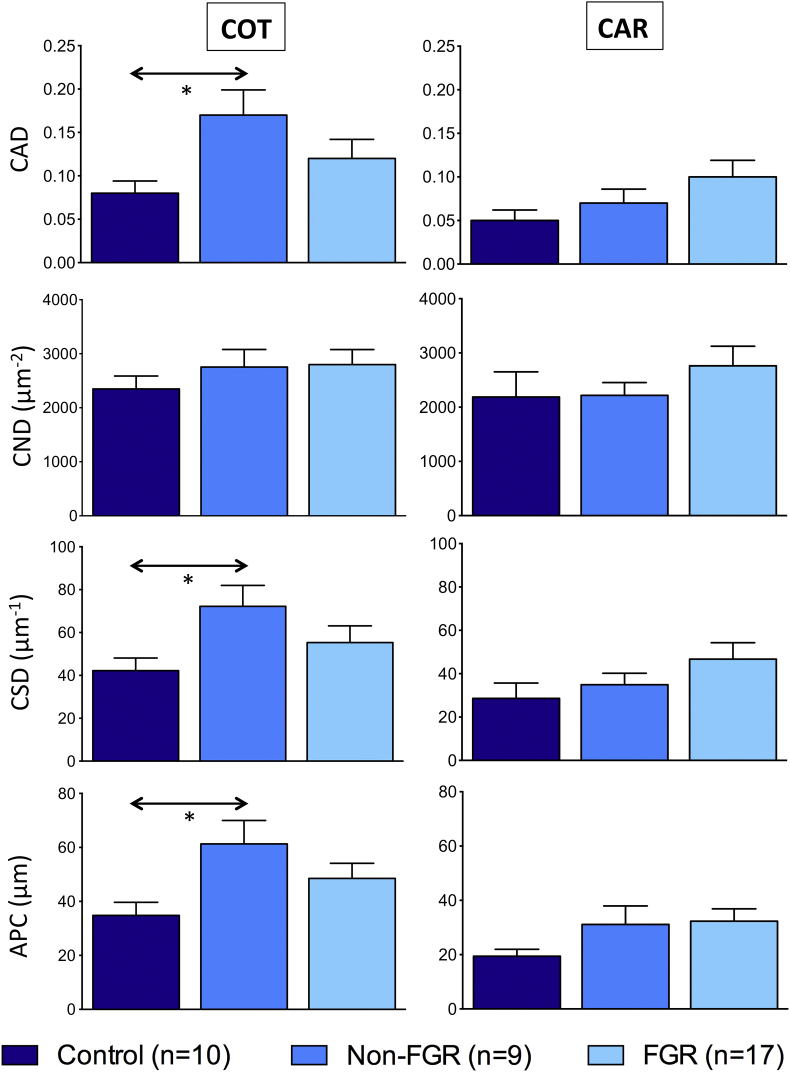
**Maternal and fetal placental vascular indices at 131 days gestation**. Placental vascular indices determined separately in maternal caruncle (CAR) and fetal cotyledon (COT) following image analysis of immunostained sections of whole placentomes sampled from adolescent sheep dams at 131 ± 0.3 days gestation who received a control intake (Control group, n = 12) or a high intake (overnourished, n = 27) of the same complete diet to induce normal fetoplacental growth or placental and fetal growth restriction (FGR), respectively. Overnourished pregnancies were further subdivided into those subsequently defined as FGR, (fetal weight >2SD below Control group mean, n = 17) or less perturbed with relatively “normal” fetal weight (Non-FGR, n = 10). Data are presented as mean ± SEM. * indicates p < 0.05 for individual post-hoc comparisons (using test of least significant difference) following one-way analysis of variances. Abbreviations: CAD = capillary area density; CND = capillary number density; CSD = capillary surface density; APC = area per capillary [see Methods for calculations].

Fig. 3 details COT/CAR placental vascular indices following image analysis of immunostained whole placentomes. Placental vascularity data could not be reliably obtained for 2/12 and 1/10 animals in Control and Non-FGR groups, respectively. There were no significant differences in any CAR indices, however three COT indices were affected by dietary intake. Irrespective of FGR status, relative to controls, overnourished pregnancies exhibited increased capillary area density (0.14 ± 0.018 vs. 0.08 ± 0.014, p = 0.017), capillary surface density (61.2 ± 6.21 vs. 42.2 ± 5.90 μm^−1^, p = 0.035) and area per capillary (53.0 ± 4.80 vs. 34.8 ± 4.86 μm^2,^, p = 0.037). Values were highest in the Non-FGR group, were all significantly greater than the Control group (p = 0.019, p = 0.028 and p = 0.014 for CAD, CSD and APC, respectively) and were intermediate in the FGR group. There were no differences between Control, Non-FGR and FGR groups in proliferation index in the CAR (1.6 ± 0.53, 2.9 ± 0.65 and 3.0 ± 0.94%; p = 0.491) or COT (6.5 ± 1.40, 9.8 ± 2.78 and 8.7 ± 1.03%; p = 0.416).

## 5 Discussion

In this study, overnourishing pregnant adolescent ewes produced FGR, defined as birthweight >2SD below that of normally-grown control-fed adolescent ewes in 63% of pregnancies [5], [25], whilst fetal growth was relatively unperturbed in the remaining (non-FGR) overnourished pregnancies. Accordingly, mean fetal weight was reduced (by 28%) in the FGR group, relative to controls, but not in the Non-FGR group. By contrast, total placental weight was significantly reduced in both FGR and Non-FGR overnourished pregnancies (by 37% and 22%, respectively) and overnourishment was associated with higher fetal:placental weight ratios, suggesting increased placental efficiency, irrespective of FGR status. A previous study found that ∼20% of ovine placentomes had to be ablated mid-gestation to significantly perturb fetal growth in late gestation [26] and our findings closely support this estimate of the functional reserve capacity of the sheep placenta. The key role of the placenta in the establishment of FGR in this paradigm is reinforced by strong correlations between fetal and placental weights. However, given the equivalent fetal:placental weight ratios in FGR and Non-FGR overnourished groups, placental mass alone does not completely explain the differential in fetal growth. There is a need to consider other aspects of placental structure/function to explain the heterogeneous response to overnourishment of pregnant adolescent ewes.

Our most important finding was that three COT vascular indices were greater in overnourished versus control-intake pregnancies. This most likely reflected an adaptive response to the experimental insult, aiming to maximise fetal nutrient supply during exponential growth in late gestation. An increase in COT vascularity, presumably also compensatory in nature, was previously observed at d90 in overnourished versus control-intake pregnancies but this difference did not persist to d130 [6]. Interestingly, previously it was only the CND parameter that was impacted whereas the present study revealed differences in CAD, CSD and APC but not CND. Failure to detect differences in late-gestation CND may simply reflect greater variability, as CND increases 10-fold from d50 to d140 [27]. Alternatively it may indicate a drive towards dilatation of existing blood vessels over angiogenesis. Irrespective, the observation that COT vascularity was greatest in the Non-FGR group is novel and suggests that compensation at a vascular level is more pronounced in pregnancies wherein fetal growth is preserved. By means of deduction, vascular adaptation in the FGR group could be considered suboptimal and may underlie subsequent retardation of fetal growth.

Despite no change in COT mRNA expression of most angiogenic ligands/receptors, the patterns observed in vascularity were mirrored by *ANGPT2*, which was upregulated by overnourishment and greatest in the Non-FGR group. Placental *ANGPT2* mRNA expression is similarly upregulated in human FGR [28] and hyperthermia-induced ovine FGR at d55 [29]. Moreover, in healthy placental villous explants, hypoxia induces *ANGPT2* expression [30], which is strongly associated with vascular remodeling and facilitates angiogenic effects of VEGF and other factors [31], [32]. Thus, COT *ANGPT2* may be a sensitive molecular marker of fetal placental vascularity.

Notwithstanding shifts in vascularity and *ANGPT2* expression, ultrasonographic placentome/UA Doppler indices did not differ between the two overnourished groups at any stage. Accordingly, values in the Non-FGR group remained perturbed relative to control-fed dams, despite relatively preserved fetal growth. The only non-fetal ultrasound parameter to differ significantly between FGR and Non-FGR groups was UCD. Clinically, a lean umbilical cord is a risk factor for adverse perinatal outcomes and reflects diminution of the Wharton's jelly and umbilical vein [33].

It can be challenging to separate cause and consequence in uteroplacental FGR. Given the insidious nature of chronic uteroplacental insufficiency, there is usually time for adaptation to occur. Fetal responses such as “brain sparing” are initiated by reduced oxygen/nutrient supply [34]. Hypoxia triggers vascular remodelling in the placenta [35] and numerous studies in this paradigm indicate an early insult on uteroplacental development [6], [7], [8], [9], [10], [11], [12] preceding the late-gestation reduction in placental mass. Switchover studies in adolescent sheep suggest that high nutritional intakes exert their strongest effects on pregnancy outcome in the middle third of gestation [36]. Moreover, fetal growth cannot be “rescued” by switching from high-to low-intake in the final third of gestation, once placental growth has been perturbed beyond the functional reserve capacity of the organ [37]. Given that the primary uteroplacental insult due to overnourishment occurs in early to mid-gestation, investigations in late gestation likely reflect both the initial pathophysiology and adaptive processes during the second half of pregnancy.

In the present study, there were no measurable changes in CAR vascularity or cell proliferation. Nevertheless, there were major reductions in several angiogenic ligands in FGR and/or Non-FGR overnourished versus control-intake pregnancies, namely *VEGF* and its receptors (*FLT1*/*KDR*) and both angiopoietins. mRNA expression of all five parameters was lowest in the FGR group and *FLT1*/*KDR*/*ANGPT1*/*ANGPT2* expression was significantly reduced compared with the Non-FGR group, in keeping with the more severe phenotype. These likely represent stigmata of the primary uteroplacental insult, as there were no apparent compensatory responses in the CAR. This agrees with our earlier study demonstrating attenuated *VEGFA/FLT1/ANGPT1*/*ANGPT2* and major reductions in UBF mid-gestation in overnourished adolescent dams [7], [11].

Collectively, our findings suggest that the placenta has an intrinsic reserve capacity that must be exceeded before FGR is established. This may explain why nutrition must be radically altered (e.g. famine) or applied to vulnerable groups (e.g. still-growing adolescents) in order to significantly perturb fetal growth. For example, overnourishment of adult ewes of the same genotype using the same diet as that provided to the adolescent dams herein is not associated with any significant perturbation of fetal growth despite an increase in adiposity, suggesting that adult sheep dams are relatively insensitive to overnutrition [38]. By contrast, high intakes have been associated with FGR and/or fetal programming effects in overnourished non-human primates, as well as human maternal obesity and/or excessive gestational weight gain [39], [40]. In the present study, it was also notable that relatively unperturbed fetuses also exhibited modified placental vascularity. This is arguably more akin to late-onset FGR in humans, wherein offspring may not be small-for-gestational-age and thus remain clinically undetected. Despite birthweights within the normal range, such individuals are at increased risk of adult-onset disease [41], thus assessment of placental structure and/or function is likely to be important, independently of fetal growth *per se*.

With respect to the effects of overnutrition on CAR indices, it remains unknown precisely what nutritional signals might potentially be read by the maternal placental tissues. The mammalian target of rapamycin (mTOR) pathway, which is likely to play a central role, can be stimulated or inhibited directly or indirectly by circulating macronutrients, cortisol, adiponectin, IGF1, leptin or insulin, or by changes in uteroplacental blood flow [42], which may explain how placental insufficiency can develop despite normal nutrient availability. It is noteworthy that maternal concentrations of glucose, insulin and IGF1 were greatest in the FGR overnourished group in the present study, in support of this assumption. It also remains unknown to what degree a potential shift in placentome type may be important. In the present study, there was a trend towards more A and less D type placentomes in the FGR group, which may represent stigmata of failed adaptation, given that evolution of A into D type placentomes is generally considered to be a process aiming to maximise the surface area available for gaseous exchange. In keeping with this theory, the proportion of A type placentomes was negatively associated with CAR APC. Furthermore, the lack of significant differences in placentome size or type between Control and Non-FGR overnourished groups suggests that the increased vascular parameters observed in the latter at a histological level would not be expected to relate to the gross assessment.

In summary, relative to control-intake sheep pregnancy, overnourished pregnancies are characterised by higher COT vascularity, which likely represents a compensatory response to reduced nutrient supply leading to enhanced placental efficiency. Relative to overnourished pregnancies in which fetal growth is relatively preserved, overnourished sheep pregnancies that culminate in marked FGR have less placental vascularity suggesting incomplete adaptation to the prenatal insult. In contrast, whilst CAR vascularity is not significantly impacted by nutritional manipulation, reduced CAR mRNA expression of a panel of angiogenic ligands/receptors is apparent in late gestation, consistent with a putative nutritionally-mediated uteroplacental insult earlier in pregnancy.

## Figures and Tables

**Table 1:** Fetal/placental biometry and umbilical artery Doppler measurements in late gestation.

Parameter	Control (n = 12)	Non-FGR (n = 10)	FGR (n = 17)	P Value
Biparietal diameter (mm)	54.7 ± 0.29^a^	53.9 ± 0.76^ab^	52.5 ± 0.43^b^	**0.004**
Abdominal circumference (mm)	286 ± 2.4^a^	270 ± 4.4^b^	259 ± 2.6^c^	**<0.001**
Femur length (mm)	59.7 ± 1.17^a^	59.3 ± 1.2^a^	54.4 ± 0.69^b^	**<0.001**
Tibia length (mm)	77.4 ± 1.07^a^	76.4 ± 1.23^a^	72.8 ± 1.11^b^	**0.012**
Renal volume (cm^3^)	10.0 ± 0.35^a^	8.8 ± 0.51^b^	7.7 ± 0.20^c^	**<0.001**
Placentome index (cm^2^)	3.7 ± 0.18^a^	2.9 ± 0.25^b^	2.6 ± 0.18^b^	**0.001**
Umbilical cord diameter (mm)	22.9 ± 0.42^a^	21.3 ± 0.59^b^	19.9 ± 0.19^c^	**<0.001**
Umbilical artery pulsatility index	0.89 ± 0.04^a^	1.12 ± 0.05^b^	1.05 ± 0.03^b^	**<0.001**
Umbilical artery resistance index	0.59 ± 0.01^a^	0.66 ± 0.02^b^	0.65 ± 0.01^b^	**0.001**
Umbilical artery systolic to diastolic ratio	2.28 ± 0.16^a^	3.04 ± 0.15^b^	2.80 ± 0.09^b^	**0.001**

P values shown are for overall ANOVA. Mean values within a row with unlike superscripts are significantly different (p < 0.05 for individual post-hoc comparisons). Data are presented as mean ± SEM.

**Table 2:** Pregnancy outcome data from necropsy in late gestation.

Parameter	Control (n = 12)	Non-FGR (n = 10)	FGR (n = 17)	P Value
Fetal weight (g)	5084 ± 124^a^	4824 ± 208^a^	3640 ± 117^b^	**<0.001**
Placentome number	113 ± 5.2^a^	107 ± 3.7^a^	93 ± 4.0^b^	**0.005**
Total placentome weight (g)	521 ± 23.8^a^	406 ± 26.9^b^	330 ± 23.8^c^	**<0.001**
Average placentome weight (g)	4.7 ± 0.21^a^	3.8 ± 0.22^b^	3.6 ± 0.21^b^	**0.002**
Membrane weight (g)	281 ± 15.9^a^	255 ± 14.9^a^	206 ± 10.6^b^	**0.001**
Total placental weight (g)	802 ± 36.1^a^	661 ± 40.1^b^	536 ± 32.8^c^	**<0.001**
Placentome size				
% <1 cm	18.3 ± 2.21	16.0 ± 1.5	18.9 ± 2.8	0.728
% 1–2 cm	16.5 ± 3.0^a^	23.6 ± 4.0^ab^	25.2 ± 2.0^b^	0.050
% 2–5 cm	62.0 ± 3.0	58.9 ± 4.6	54.6 ± 3.7	0.367
% >5 cm	3.1 ± 0.8	1.4 ± 0.6	1.3 ± 0.6	0.114
Placentome type				
% A type	33.8 ± 8.9	36.7 ± 7.3	55.4 ± 7.5	0.109
% B type	39.8 ± 5.7	40.8 ± 5.9	33.2 ± 6.1	0.621
% C type	11.4 ± 3.1	5.2 ± 3.8	5.5 ± 2.1	0.251
% D type	15.2 ± 3.8	17.8 ± 9.2	6.2 ± 2.5	0.209
Placental efficiency (g fetus/g placenta)	9.9 ± 0.42^a^	12.1 ± 0.53^b^	11.7 ± 0.62^b^	**0.033**
Male-to-female fetal sex ratio	7:5	4:6	7:10	**0.595**

P values shown are for overall ANOVA with the single exception of the male-to-female sex ratio, for which the Chi square test was employed – bold indicates p < 0.05. Mean values within a row with unlike superscripts are significantly different (p < 0.05 for individual post-hoc comparisons). Data are presented as mean ± SEM.

**Table 3:** Maternal caruncular and fetal cotyledonary mRNA expression of angiogenic ligands/receptors and glucose transporters in late gestation placentomes.

Placental compartment and gene of interest		Control (n = 12)	Non-FGR (n = 10)	FGR (n = 17)	P value
Maternal caruncle	*VEGFA*	27.3 ± 3.04^a^	23.6 ± 7.45^ab^	18.3 ± 1.49^b^	**0.014**
	*FLT1*	18.8 ± 2.26^a^	17.3 ± 5.46^a^	9.6 ± 1.13^b^	**0.002**
	*KDR*	23.8 ± 2.40^a^	21.3 ± 6.72^a^	15.1 ± 1.40^b^	**0.009**
	*NOS3*	28.6 ± 2.82	39.6 ± 12.52	29.9 ± 2.87	0.085
	*FGF2*	15.7 ± 1.54	14.3 ± 4.51	11.5 ± 1.27	0.154
	*ANGPT1*	24.0 ± 1.62^a^	25.2 ± 7.96^a^	17.1 ± 1.44^b^	**0.004**
	*ANGPT2*	14.0 ± 1.17^a^	14.9 ± 4.72^a^	10.9 ± 0.70^b^	**0.010**
	*TEK*	21.6 ± 2.63	20.2 ± 6.40	19.6 ± 1.76	0.785
	*GUCY1*	16.5 ± 1.52	13.3 ± 4.20	13.8 ± 1.34	0.276
	*SLC2A1*	18.6 ± 1.14^a^	19.0 ± 2.05^a^	13.3 ± 0.84^b^	**0.003**
	*SLC2A3*	13.2 ± 0.81^a^	10.4 ± 0.89^b^	9.3 ± 0.68^b^	**0.003**
Fetal cotyledon	*VEGFA*	26.5 ± 3.53	24.3 ± 7.69	34.7 ± 3.06	0.082
	*FLT1*	15.1 ± 2.42	11.0 ± 3.48	16.7 ± 1.77	0.177
	*KDR*	17.0 ± 2.44	13.5 ± 4.26	21.4 ± 2.12	0.096
	*NOS3*	16.1 ± 2.17	15.4 ± 4.87	22.1 ± 2.56	0.075
	*FGF2*	9.9 ± 1.07	11.6 ± 3.65	11.2 ± 0.86	0.510
	*ANGPT1*	21.9 ± 1.64	23.9 ± 7.56	22.7 ± 0.90	0.654
	*ANGPT2*	13.0 ± 0.79^a^	18.2 ± 5.76^b^	16.2 ± 0.92^b^	**0.008**
	*TEK*	14.0 ± 0.79	15.5 ± 4.91	16.6 ± 0.73	0.101
	*GUCY1*	8.9 ± 0.90	8.1 ± 2.56	8.5 ± 0.45	0.735
	*SLC2A1*	22.9 ± 1.68	25.1 ± 1.57	23.7 ± 1.00	0.568
	*SLC2A3*	20.7 ± 1.31	20.8 ± 1.00	21.6 ± 0.92	0.800

P values shown are for overall ANOVA – bold indicates p < 0.05. Mean values within a row with unlike superscripts are significantly different (p < 0.05 for individual post-hoc comparisons). Data are presented as mean ± SEM. Abbreviations: *VEGFA* = vascular endothelial growth factor; *FLT1* = fms-related tyrosine kinase 1 (VEGF receptor 1), *KDR* = kinase insert domain receptor (VEGF receptor 2); *NOS3* = nitric oxide synthase 3; *FGF2* = fibroblast growth factor 2; *ANGPT1* = angiopoietin 1; *ANGPT2* = angiopoietin 2; *TEK* = endothelial tyrosine kinase; *GUCY1* = soluble guanylate cyclase (nitric oxide receptor); *SLC2A1* = solute carrier family 2 (facilitated glucose transporter) member 1; *SLC2A3* = solute carrier family 2 (facilitated glucose transporter) member 3. All data presented are expressed relative to the housekeeping gene 18S.
